# *In Vitro* Study on Glucose Utilization Capacity of Bioactive Fractions of *Houttuynia cordata* in Isolated Rat Hemidiaphragm and Its Major Phytoconstituent

**DOI:** 10.1155/2016/2573604

**Published:** 2016-01-26

**Authors:** Manish Kumar, Satyendra K. Prasad, Siva Hemalatha

**Affiliations:** ^1^Pharmacognosy Research Laboratory, Department of Pharmaceutics, Indian Institute of Technology (Banaras Hindu University), Varanasi 221005, India; ^2^Department of Pharmaceutical Sciences, R. T. M. Nagpur University, Nagpur 440033, India

## Abstract

*Objective*. The whole plant of* Houttuynia cordata* has been reported to have potent antihyperglycemic activity. Therefore, the present study was undertaken to investigate the glucose utilization capacity of bioactive fractions of ethanol extract of* Houttuynia cordata* (HC) in isolated rat hemidiaphragm.* Methods*. All the fractions, that is, aqueous (AQ), hexane (HEX), chloroform (CHL), and ethyl acetate (EA), obtained from ethanol extract of* H. cordata* were subjected to phytochemical standardization use in quercetin as a marker with the help of HPTLC. Further, glucose utilization capacity by rat hemidiaphragm was evaluated in 12 different sets of* in vitro* experiments. In the study, different fractions from* H. cordata* as mentioned above were evaluated, where insulin was used as standard and quercetin as a biological standard.* Results*. Among all the tested fractions, AQ and EA significantly increased glucose uptake by isolated rat hemidiaphragm compared to negative control. Moreover, AQ fractions enhanced the uptake of glucose significantly (*p* < 0.05) and was found to be more effective than insulin.* Conclusions*. The augmentation in glucose uptake by hemidiaphragm in presence of AQ and EA fractions may be attributed to the presence of quercetin, which was found to be 7.1 and 3.2% w/w, respectively, in both the fractions.

## 1. Introduction

Diabetes mellitus is a group of metabolic disorders, characterized by hyperglycemia resulting from the defects in insulin secretion, insulin action, or both [1]. Numbers of therapies have been used to improve the status of diabetes by different mechanisms such as inhibition of carbohydrate metabolizing enzymes, manipulation of glucose transporters, *β*-cell regeneration, and enhancing the insulin releasing activity [2]. Although oral hypoglycemic agents and insulin are the cornerstones of treatment of diabetes and are effective in controlling hyperglycemia, they have prominent side effects and many limitations exist in their use [3]. Therefore, the management of diabetes without any side effect is still a challenge to the medical system. Many efforts have been made to identify new hypoglycemic agents obtained from different sources especially from medicinal plants because of their effectiveness, fewer side effects, and relatively low cost. Several medicinal plants have been investigated for their beneficial use in different types of diabetes in the traditional system of medicine; however, studies related to their biologically active components are still lacking and require a great encouragement [4].

*Houttuynia cordata* Thunb. (Saururaceae) is a single species of its genus and is native to Japan, South-East Asia, and Himalayas. The whole plant of* H. cordata* is being widely used as a medicinal salad in North-East region of India for lowering the blood glucose level and is commonly known by the name Jamyrdoh [5]. Traditionally it is used to cure various human ailments throughout South-East Asia, namely, cancer, coughs, dysentery, enteritis, fever, snake bites, and skin disorders. Pharmacologically this plant has been validated for antioxidant, antihypertension, antiedema, anti-inflammatory, antipyretic, antipurulent, antihyperglycemic, and aldose reductase inhibitory activities [6–9]. The major active constituents of* H. cordata* include quercetin, chlorogenic acid, caffeic acid, hyperin, and rutin which have exhibited antioxidant, antihyperglycemic, anticancer, and neuroprotective effects in various experimental models [10–15]. With these reports, it was suggested that flavonoids are molecules capable to interact with more than one target and therefore termed as privileged structures in accordance with Patchett's definition [16]. Conventional antidiabetic agents can affect several pathways of glucose metabolism such as insulin secretion, glucose uptake by target organs, and nutrient absorption. As the plant has been reported for its antihyperglycaemic potential; therefore, estimation of glucose content in rat hemidiaphragm may act as a reliable method for determining the efficiency of the plant in* in vitro* peripheral uptake of glucose. Therefore, on the basis of above background the present study was undertaken for the first time to assess the potential role of different fraction of* H. cordata* on the peripheral utilization of glucose.

## 2. Material and Methods

### 2.1. Plant Material

Whole plant of* H. cordata* was collected during the months of June–September (2012) from various areas of the West and East Jaintia Hills district (namely, Jowai, Mihmyntdu, Khliehriat, and Ladrymbai) of Meghalaya, North-East, India. Voucher herbarium specimen (COG/HC/011-2012) was prepared and preserved along with sample of crude drug in the Pharmacognosy research laboratory of Department of Pharmaceutics, Indian Institute of Technology, Banaras Hindu University, Varanasi (UP), India. The plant material was identified and authenticated by Dr. B. K. Sinha (Scientist In-charge), Botanical Survey of India, Shillong, Meghalaya.

### 2.2. Preparation and Standardization of Extract and Its Fractions

Whole plant of* H. cordata* was washed with water, shade dried, and ground in a mill and was passed through sieve #40 to obtain a homogenous powder. The coarsely powdered plant material (1 Kg) of* H. cordata* was exhaustively extracted for 24 h by soxhlation using 95% ethanol (3 L v/v) as solvent for extraction. The resulting extract was filtered and concentrated under reduced pressure to obtain the crude ethanol extract of* H. cordata* (EHC) (yield: 13.2% w/w). The ethanol extract was then subjected to successive fractionation by suspending in aqueous media and then partitioning with solvents of varying polarity such as hexane, chloroform, and ethyl acetate in order of their ascending polarity.

Further, all the fractions, that is, aqueous (AQ), hexane (HEX), chloroform (CHL), and ethyl acetate (EA), from* H. cordata* were standardized with quercetin (QC) using high performance thin layer chromatography (HPTLC). Mobile phase for developing the chromatogram was composed of chloroform : methanol and formic acid mixture in the ratio 7.5 : 1.5 : 1 (v/v/v). The concentration of quercetin was taken as 0.5 mg/mL, while that of the fractions was taken as 5 mg/mL in methanol. The study was carried out using Camag-HPTLC instrumentation (Camag, Mutten, Switzerland) equipped with Linomat V sample applicator, Camag TLC scanner 3, Camag TLC visualizer, and WINCATS 4 software for data interpretation. The *R*_*f*_ values were recorded and the developed plate was screened and photodocumented at ultra violet range with wavelength (*λ*_max_) of 254 nm (details in Supplementary data; see Supplementary Material available online at http://dx.doi.org/10.1155/2016/2573604).

### 2.3. Study of Glucose Utilization by Isolated Rat Hemidiaphragm Technique

Glucose uptake by rat hemidiaphragm was estimated by the method described by Hemalatha et al. [17], using regular insulin (Biocon Ltd.) as a positive control group and rat's hemidiaphragm for the assay. Glucose uptake per gram of tissue was calculated as the difference between the initial and final glucose content in the incubated medium [18].

### 2.4. Animals

Albino rats of Charles foster strain with body weights of 160–200 g were obtained from the Central Animal House (Reg. number 542/02/ab/CPCSEA), Institute of Medical Science (IMS), Banaras Hindu University (BHU), Varanasi, India. Rats were fed with normal laboratory pellet diet (Hindustan lever Ltd., India) with water* ad libitum* and were housed in polypropylene cages under standard laboratory condition [12 h light/12 h darkness, (21 ± 2°C)]. The experimental protocol has been approved by the institutional animal ethics committee (Reference number Dean/10-11/58 dated 07.03.2011).

### 2.5. Assessment of Glucose Utilization by Rat Hemidiaphragm Method

For the estimation of glucose utilization by rat hemidiaphragm, twelve sets containing three numbers of graduated test tubes (*n* = 3) each were taken. Group I was served as a control which contained 2 mL of Tyrode's solution with 2% glucose and group II contained 2 mL Tyrode's solution with 2% glucose and regular insulin (Biocon Ltd.), that is, 1 mL of 0.25 IU/mL of solution. Group III contained 2 mL of Tyrode's solution with 2% glucose and 1 mL of (1 mg/mL) solution of quercetin, used as biological standard. Group IV contained 2 mL of Tyrode's solution with 2% glucose and regular insulin (1 mL of 0.25 IU/mL solution) and 1 mL of (1 mg/mL) solution of quercetin. Group V contained 2 mL of Tyrode's solution with 2% glucose and 1 mL of (25 mg/mL) of AQ fraction. Group VI contained 2 mL of Tyrode's solution with 2% glucose and 1 mL of (25 mg/mL) of AQ fraction and regular insulin (1 mL of 0.25 IU/mL solution). Group VII contained 2 mL of Tyrode's solution with 2% glucose and 1 mL of (25 mg/mL) of EA fraction. Group VIII contained 2 mL of Tyrode's solution with 2% glucose and 1 mL of (25 mg/mL) of EA fraction and regular insulin (1 mL of 0.25 IU/mL solution). Group IX contained 2 mL of Tyrode's solution with 2% glucose and 1 mL of (25 mg/mL) of CHL fraction. Group X contained 2 mL of Tyrode's solution with 2% glucose and 1 mL of (25 mg/mL) of CHL fraction and regular insulin (1 mL of 0.25 IU/mL solution). Group XI contained 2 mL of Tyrode's solution with 2% glucose and 1 mL of (25 mg/mL) of HEX fraction. Group XII contained 2 mL of Tyrode's solution with 2% glucose and 1 mL of (25 mg/mL) of HEX fraction and regular insulin (1 mL of 0.25 IU/mL solution).

The final volume of all the test tubes was made up to 4 mL with distilled water to match the volume of the test tubes of group IV. Albino rats were sacrificed by cervical dislocation after overnight fasting. The diaphragms were dissected out quickly with minimal strain and divided into two equal halves. Two diaphragms from the same animal were not used for the same set of experiment. The hemidiaphragms were placed in test tubes and incubated for 30 min at 37°C in an atmosphere of 95% oxygen and 5% CO_2_ with shaking at 140 cycles/min. Glucose uptake per gram of tissue was calculated as the difference between the initial and final glucose content in the incubated medium.

### 2.6. Statistical Analysis

The data were analyzed with GraphPad Prism version 5 (San Diego, CA). Statistical analysis was done by one-way ANOVA, followed by Tukey's multiple comparison test. Data are expressed as mean ± SEM. A level of *p* < 0.05 was accepted as statistically significant.

## 3. Result

**Figure 1:**
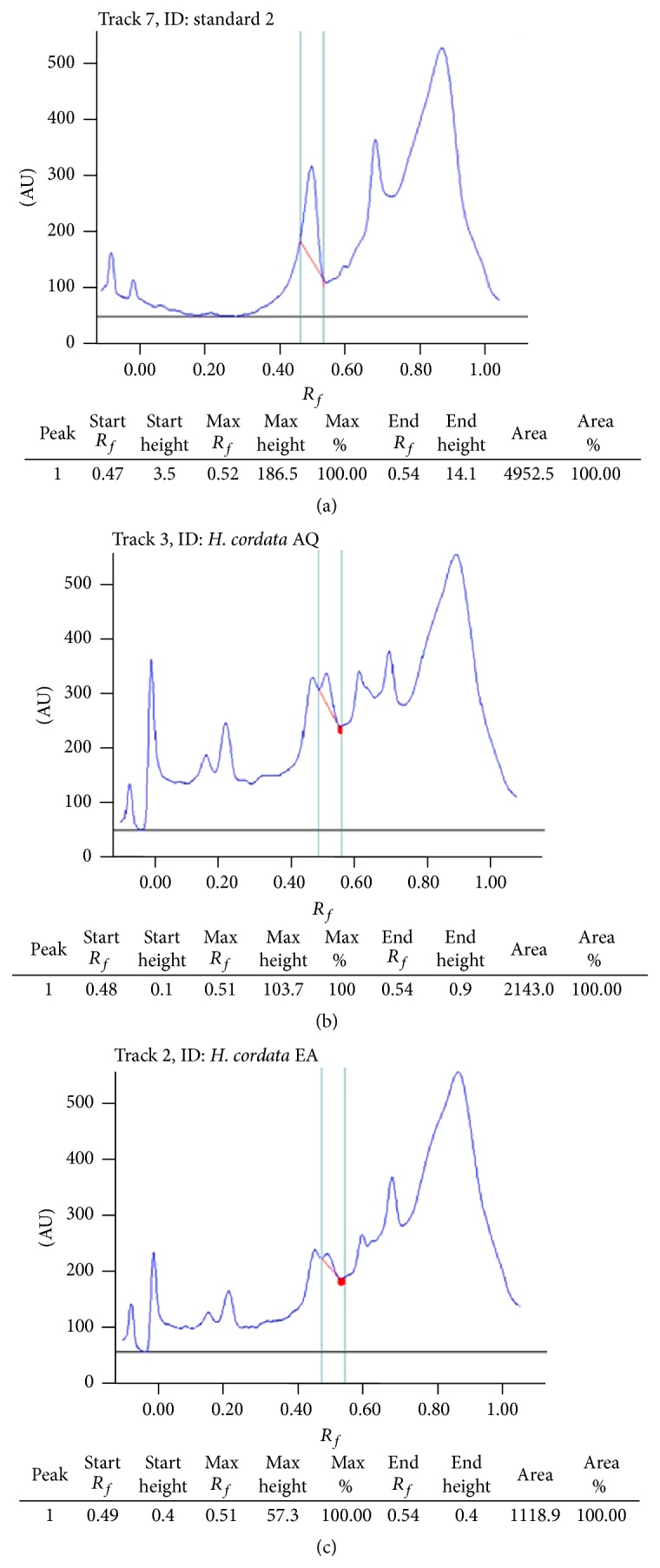
HPTLC chromatograms of quercetin (a); HPTLC chromatograms of quercetin in AQ fraction of* H. cordata* (b); HPTLC chromatograms of quercetin in EA fraction of* H. cordata* (c).

Different concentrations of fractions and quercetin were subjected to HPTLC analyses using mobile phase as chloroform : methanol and formic acid in the ratio 7.5 : 1.5 : 1 (v/v/v). *R*_*f*_ value of quercetin was reported to be 0.48 and the quercetin content was found to be 7.1% and 3.2% w/w in AQ and EA fractions, respectively, whereas CHL and HEX fractions did not show any measurable quercetin content (Figures 1(a), 1(b), and 1(c)).

In the present study different fractions from* H. cordata,* namely, aqueous, ethyl acetate, chloroform, and hexane fraction, were estimated for glucose utilization capacity by isolated rat hemidiaphragm. Among all the tested fractions, AQ fractions enhanced the uptake of glucose by isolated rat hemidiaphragm significantly (*p* < 0.05), which was found to be more effective than insulin (Table 1). Moreover, quercetin which was used as biological marker also showed significant glucose uptake (*p* < 0.05) when compared to glucose uptake by rat hemidiaphragm of control group (Tyrode's solution with 2% glucose + rat hemidiaphragm) only (Table 1).

## 4. Discussion

Several investigations have demonstrated the beneficial effects of* H. cordata*. In our previous studies ethanol extract of* H. cordata* has been shown to possess antihyperglycemic activity, increase the antioxidant status of pancreatic *β*-cells, and promote the insulin secretion in rodents [8]. In the present study, we have made an attempt to investigate the plausible mechanism of action for the above proposed activity. From the literature search it was found that many herbs and plant products have been shown to have antidiabetic action due to high flavonoids contents, which are known to be bioactive antidiabetic principles. The positive results of the* in vivo* study that was undertaken previously by us in rodents for antidiabetic potential of* H. cordata* may be due the presence of flavonoid compounds.

The estimation of glucose content in rat hemidiaphragm was employed for* in vitro* study of peripheral uptake of glucose. The results showed that the AQ and EA fractions significantly increased glucose uptake by isolated rat hemidiaphragm as compared to normal control (Table 1). Furthermore, the AQ fractions enhanced the uptake of glucose significantly (*p* < 0.05) and were found to be more effective than insulin. Administration of QC significantly (*p* < 0.05) enhanced the uptake of glucose compared to control group as observed in insulin treated group. However, there was no significant difference observed when AQ fractions and insulin administered groups were together compared to AQ fraction alone. It is reported that insulin stimulates glucose transport in the isolated rat diaphragm primarily through a translocation of functional glucose transport units from an intracellular membrane pool to the plasma membrane [19]. As we have discussed earlier* H. cordata* enhances the insulin secretion; therefore it may be one of the rationale for glucose utilization by rat hemidiaphragm.

It is interesting to note that the quercetin content in AQ and EA fractions was significant as compared to CL and HEX (Figures 1(a), 1(b), and 1(c)), where it was not detected.* H. cordata* has been previously reported for the presence of a number of phenolic compounds such as quercitrin, afzelin, hyperin, rutin, quercetin, chlorogenic acid, and caffeic acid. However, amongst them, quercetin and rutin have been reported to possess action towards increase in glucose uptake by rat hemidiaphragm [20]. The results from the present study revealed that the augmentation in glucose utilization by hemidiaphragm in presence of AQ fractions may be attributed to the presence of quercetin, as it represents the major component of these fractions. However, the role of other phytoconstituents cannot be discounted unless verified by further experiments.

## 5. Conclusion

In the present study, glucose utilization capacity of different fractions of* H. cordata* was carried out on isolated rat hemidiaphragm. Among the tested fractions, aqueous fraction showed increased utilization of the glucose by hemidiaphragm, which may be attributed to the presence of high water soluble flavonoid contents. These results suggest that* H. cordata* had some extra pancreatic mechanism like glucose uptake by peripheral tissues.

## Supplementary Material

The developed HPTLC plate was screened and photo-documented at ultra violet range with wavelength (λmax) of 254 nm. The plate was developed using Camag- HPTLC instrumentation (Camag, Mutten, Switzerland) equipped with Linomat V sample applicator, Camag TLC scanner 3, Camag TLC visualizer. (details in Supplementary data).

## Figures and Tables

**Table 1:** Effect of different fractions of *H. cordata* on glucose utilization by isolated rat hemidiaphragm.

Group	Incubation medium	Glucose uptake (mg/g/30 min)
Control	Tyrode solution with glucose (2%)	15.74 ± 0.38
Insulin	Tyrode solution with glucose (2%) + insulin (0.25 IU/mL)	29.65 ± 0.54^a^
QC	Tyrode solution with glucose (2%) + quercetin (1 mg/mL)	31.42 ± 1.87^a^
Insulin + QC	Tyrode solution with glucose (2%) + insulin (0.25 IU/mL) + quercetin (1 mg/mL)	34.10 ± 1.03^a^
AQ	Tyrode solution with glucose (2%) + aqueous fraction (25 mg/mL)	35.26 ± 0.52^a,b^
Insulin + AQ	Tyrode solution with glucose (2%) + insulin (0.25 IU/mL) + aqueous fraction (25 mg/mL)	36.21 ± 1.63^a,b^
EA	Tyrode solution with glucose (2%) + ethyl acetate fraction (25 mg/mL)	24.58 ± 0.56^a^
Insulin + EA	Tyrode solution with glucose (2%) + insulin (0.25 IU/mL) + ethyl acetate fraction (25 mg/mL)	28.60 ± 0.33^a^
CHL	Tyrode solution with glucose (2%) + chloroform fraction (25 mg/mL)	19.32 ± 0.84
Insulin + CHL	Tyrode solution with glucose (2%) + insulin (0.25 IU/mL) + chloroform fraction (25 mg/mL)	23.33 ± 0.54^a^
HEX	Tyrode solution with glucose (2%) + hexane fraction (25 mg/mL)	17.41 ± 0.81
Insulin + HEX	Tyrode solution with glucose (2%) + insulin (0.25 IU/mL) + hexane fraction (25 mg/mL)	22.32 ± 0.36^a^

All results are expressed as mean ± SEM. Statistical comparison was determined by one-way ANOVA followed by Tukey's multiple comparison test. ^a^ *p* < 0.05, statistically significant compared to control group; ^b^ *p* < 0.05 compared to insulin.

AQ: aqueous fraction, EA: ethyl acetate fraction, CHL: chloroform fraction, and HEX: hexane fraction of *H. cordata*.
