# Supplementary figures and images for: In Vitro Study on Glucose Utilization Capacity of Bioactive Fractions of Houttuynia cordata in Isolated Rat Hemidiaphragm and Its Major Phytoconstituent
Source: Adv Pharmacol Sci. 2016 Jan 26;2016:2573604. doi: 10.1155/2016/2573604 (PMC4746272; doi:10.1155/2016/2573604)

**FIGURES**


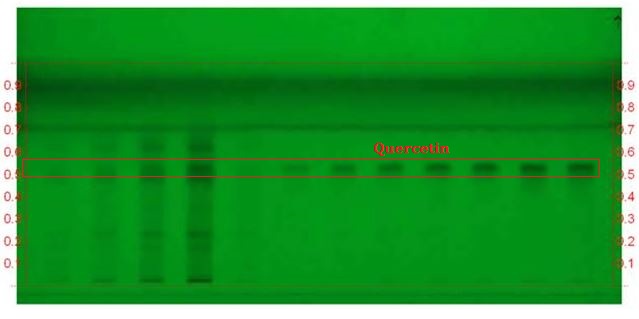
**Figure**. HPTLC fingure print of *Houttynia cordata* Thunb. at λmax 254 nm.

Supplement: Supplementary file 1 — The developed HPTLC plate was screened and photo-documented at ultra violet range with wavelength (λmax) of 254 nm. The plate was developed using Camag- HPTLC instrumentation (Camag, Mutten, Switzerland) equipped with Linomat V sample applicator, Camag TLC scanner 3, Camag TLC visualizer. (details in Supplementary data). [file 2573604.f1.docx]
